# An Improved Method for Establishing Accurate Water Potential Levels at Different Temperatures in Growth Media

**DOI:** 10.3389/fmicb.2017.01497

**Published:** 2017-08-11

**Authors:** Iqbal S. Aujla, Timothy C. Paulitz

**Affiliations:** ^1^Department of Plant Pathology, Washington State University Pullman, WA, United States; ^2^Department of Plant Pathology, Punjab Agricultural University Ludhiana, India; ^3^Wheat Health, Genetics, and Quality Research (USDA-ARS) Pullman, WA, United States

**Keywords:** NaCl, KCl, polyethylene glycol, potato dextrose broth, potato dextrose agar, water potential, temperature, interactions

## Abstract

NaCl, KCl, or PEG (polyethylene glycol)-amended potato dextrose broth (PDB), and potato dextrose agar (PDA) are essential for pure culture studies of water stress on fungi. Direct information on the actual water potential (WP) of this salt-amended PDB and PDA is lacking. Much fungal research in the past calculated WP of these salt-amended growth media by adding the WP of their constituents taken from individual salt dilution studies. But the WP of any complex solution will be modified by the level of synergism between its solutes. This study presents evidence of change in NaCl concentration due to synergism for attaining the same level of WP in NaCl solution, and NaCl amended PDB and PDA. The relation between WP and temperature and WP and salt concentration is also modified depending on the number of solutes in a growth medium. The WP of PEG-amended PDB increases with rising temperature, while that of NaCl/KCl amended PDB and PDA decreases with the increase of temperature. These results can be useful for doing pure culture studies on the biology and modeling the growth of air, water, and soil-borne fungi important in the food and agriculture industry and in terrestrial and aquatic ecosystems.

## Introduction

Temperature and moisture are the major physical factors determining the biology of any microorganism in its environment. Due to the ease of working with airborne microorganisms directly on their food substrate (host), and availability of more sensitive instrumentation for measuring environmental parameters influencing their biology, the advances in understanding the biology, and epidemiology of airborne microorganisms is much more advanced than for fungi residing in soils. The difficulty of measuring water potential (WP) in a heterogeneity of microenvironments in the soil makes it imperative to conduct pure culture studies of soil-borne microorganisms under controlled conditions for understanding their activity and how it is influenced by the environment.

Pure culture studies need a growth medium which supports the growth of a microorganism. For fungi, potato dextrose broth (PDB), and potato dextrose agar (PDA) are the most commonly used liquid and solid growth media to study colony morphology, biomass production, reproduction, survival and production of chemicals such as antibiotics. PDB is composed of extracts from potato, (including potato starch) and dextrose (glucose). PDA requires the addition of agar to the constituents of PDB.

In pure culture studies, the effect of temperature can be explored with the use of temperature controlled incubators. For studying the effect of moisture on the biology of these fungi, the growth media need amendment with different salts to explore the impact of various components of WP, a measure of the availability of moisture (Harris, 1981). Water potential is a measure of the potential energy (per unit mass or volume) of water at a point in a system relative to the potential energy of pure, free water. It is measured in megapascal (MPa) or kilopascal (kPa), (1 MPa = 1,000 kPa) and its value is always negative for any solution, as pure, free water is usually assigned the maximum value of zero MPa. The value of WP will determine the availability of water for any organism (Papendick and Campbell, 1981).

The two primary components of WP influencing water availability for a microorganism in soil are osmotic and matric component of WP. Osmotic potential is determined by the number and quantity of solutes dissolved in the water, and matric potential by the interaction of water with solids or polymers (Papendick and Campbell, 1981). In the laboratory, growth media amended with various solutes like NaCl, KCl, sucrose, and glycerol are used to study the osmotic component, and polyethylene glycol (PEG) of different molecular weights are used to examine the matric part of WP (Harris, 1981; Steuter et al., 1981). It is important to study the osmotic component by using more than one type of salt-amended growth media to establish that the effect observed is only due to WP, not due to specific solute factors (Cook and Christen, 1976).

Many studies on fungal growth have been performed on salt-amended PDB and PDA (Cook et al., 1972; Cook and Christen, 1976). The weight of salts to be added in PDB and PDA to achieve different WP levels were based on the studies done by Lang (1967) (NaCl), Campbell and Gardner (1971) (KCl), and Michel and Kaufmann (1973) (PEG-8000) on single solute dissolved in water. PEG-8000 hereafter will be referred to as PEG.

In most of these fungal growth studies done in last few decades, the WP of salt-amended PDB and PDA has been calculated by just summing up the WP of individual components of these growth media—salt (NaCl, KCl, or PEG), and PDB with or without agar. But Michel (1983) has clearly stated that the whole WP of any solution involving more than one solute is not a simple addition of WP of the individual components. Its value will be altered depending on the synergism between its solutes, which can be either positive or negative. Both PDB and PDA are not even a mixture of two simple solutes in a solution. They involve complex molecules such as PDB and agar. PDB further has potato starch and dextrose as its components. It is evident that the actual WP of salt-amended PDB and PDA should also be altered due to the similar interaction between not only PDB and agar but also with the properties of salt used. Scientific literature is lacking this evidence.

Lang (1967), Campbell and Gardner (1971), and Michel and Kaufmann (1973) have also clearly documented in their studies that at the same concentration level of NaCl, KCl, and PEG, the WP varies with the change in temperature. But no literature is available on the similar impact of temperature on PDB and PDA amended with NaCl, KCl, or PEG. In addition, more accurate technology, such as the WP4C Dew Point Hygrometer with a broader wet to dry range and higher accuracy is also available for measuring WP in the lab.

With advancements in the technology of measuring WP, this study was conducted with the objective of not only deciphering the actual WP of PDB and PDA at different concentration levels of NaCl, KCl, and PEG but also the impact of temperature. The other objective of this study was to determine the nature of interactions between NaCl, PDB, and agar.

## Materials and methods

The quantity of salt (NaCl/KCl/PEG) weight given in Tables 1–4 were used to amend PDB and PDA to standardize a WP range of 0 to −9 MPa with an interval of −1 MPa at 20°C. The target WP was checked on WP4C PotentiaMeter (METER Group, Inc. USA) after autoclaving the salt-amended PDB and PDA. The WP of the same salt and basal growth media combinations standardized at 20°C was also determined at 7, 15, 25, 30, and 35°C to test the influence of temperature. PEG mentioned in this study refers to PEG with a molecular weight of 8,000.

**Table 1:** Water potential (MPa) of PEG-8000 amended potato dextrose broth (PEG+PDB).

**Target WP**	**PEG conc. (g/l PDB^a^)**	**7°C**	**15°C**	**20°C**	**25°C**	**30°C**	**35°C**
0	0.00	−0.26 (0.01)	−0.47 (0.01)	−0.49 (0.01)	−0.32 (0.01)	−0.36 (0.01)	−0.42 (0.02)
−1	226.28	−1.01 (0.01)	−1.15 (0.13)	−1.06 (0.09)	−0.99 (0.09)	−0.93 (0.01)	−0.81 (0.09)
−2	344.00	−2.20 (0.02)	−2.07 (0.09)	−1.91 (0.02)	−1.74 (0.03)	−1.68 (0.02)	−1.47 (0.03)
−3	439.04	−3.38 (0.02)	−3.32 (0.08)	−2.91 (0.02)	−2.66 (0.03)	−2.53 (0.01)	−2.26 (0.03)
−4	520.04	−4.57 (0.03)	−4.53 (0.03)	−4.12 (0.08)	−3.64 (0.02)	−3.44 (0.02)	−3.21 (0.03)
−5	584.00	−5.61 (0.01)	−5.42 (0.08)	−5.01 (0.01)	−4.47 (0.01)	−4.22 (0.03)	−3.97 (0.01)
−6	650.20	−6.84 (0.03)	−6.43 (0.04)	−5.99 (0.04)	−5.41 (0.03)	−5.11 (0.04)	−4.74 (0.03)
−7	713.20	−8.06 (0.03)	−7.68 (0.02)	−7.02 (0.03)	−6.48 (0.03)	−6.03 (0.04)	−5.64 (0.03)
−8	768.20	−9.12 (0.03)	−8.61 (0.09)	−8.01 (0.06)	−7.41 (0.05)	−6.89 (0.03)	−6.43 (0.03)
−9	824.60	−10.22 (0.02)	−9.56 (0.05)	−9.03 (0.05)	−8.26 (0.06)	−7.72 (0.09)	−7.20 (0.05)

*WP values in each temperature column represent mean and (SD) measured by WP4C*.

a *PDB added at 24 g/1,000 ml water at 20°C*.

**Table 2:** Water potential (MPa) of NaCl amended potato dextrose broth (NaCl+PDB).

**Target WP**	**NaCl conc. (g/l PDB^a^)**	**7°C**	**15°C**	**20°C**	**25°C**	**30°C**	**35°C**
0	0.00	−0.26 (0.01)	−0.47 (0.01)	−0.49 (0.01)	−0.32 (0.01)	−0.36 (0.01)	−0.42 (0.02)
−1	8.76	−0.96 (0.01)	−0.98 (0.14)	−1.00 (0.04)	−1.02 (0.07)	−1.02 (0.07)	−1.04 (0.07)
−2	21.16	−1.81 (0.06)	−1.89 (0.06)	−1.91 (0.05)	−1.93 (0.03)	−1.95 (0.02)	−1.96 (0.05)
−3	33.64	−2.74 (0.03)	−2.85 (0.09)	−2.92 (0.05)	−2.91 (0.02)	−3.01 (0.02)	−3.01 (0.04)
−4	45.08	−3.63 (0.02)	−3.96 (0.09)	−3.86 (0.09)	−3.87 (0.02)	−3.95 (0.02)	−3.96 (0.08)
−5	57.84	−4.60 (0.04)	−4.90 (0.02)	−4.96 (0.03)	−4.95 (0.02)	−5.03 (0.01)	−5.12 (0.05)
−6	69.52	−5.54 (0.04)	−5.82 (0.02)	−5.97 (0.06)	−5.99 (0.02)	−6.10 (0.04)	−6.18 (0.01)
−7	81.36	−6.49 (0.02)	−6.88 (0.05)	−6.96 (0.03)	−7.06 (0.04)	−7.18 (0.03)	−7.28 (0.01)
−8	91.64	−7.41 (0.02)	−7.74 (0.04)	−7.92 (0.05)	−7.98 (0.02)	−8.11 (0.04)	−8.29 (0.06)
−9	103.16	−8.30 (0.03)	−8.78 (0.15)	−9.01 (0.08)	−9.02 (0.02)	−9.22 (0.03)	−9.41 (0.03)

*WP values under each temperature column represent mean and (SD) measured by WP4C*.

a *PDB added at 24 g/1,000 ml water at 20°C*.

**Table 3:** Water potential (MPa) of NaCl amended potato dextrose agar (NaCl+PDB+agar).

**Target WP**	**NaCl conc. (g/l PDA^a^)**	**7°C**	**15°C**	**20°C**	**25°C**	**30°C**	**35°C**
0	0.00	−0.31 (0.01)	−0.55 (0.07)	−0.55 (0.01)	−0.46 (0.01)	−0.46 (0.04)	−0.50 (0.05)
−1	11.95	−0.88 (0.07)	−1.24 (0.10)	−1.01 (0.05)	−1.11 (0.04)	−1.40 (0.04)	−1.48 (0.04)
−2	26.03	−1.99 (0.04)	−2.22 (0.12)	−2.01 (0.06)	−2.31 (0.05)	−2.48 (0.05)	−2.68 (0.04)
−3	37.36	−2.85 (0.01)	−2.98 (0.05)	−3.00 (0.07)	−3.22 (0.03)	−3.45 (0.04)	−3.64 (0.05)
−4	49.02	−3.77 (0.02)	−3.88 (0.10)	−4.04 (0.07)	−4.20 (0.02)	−4.56 (0.02)	−4.76 (0.09)
−5	58.99	−4.53 (0.02)	−4.72 (0.11)	−4.96 (0.04)	−5.10 (0.07)	−5.47 (0.05)	−5.58 (0.07)
−6	71.83	−5.56 (0.03)	−5.75 (0.10)	−6.03 (0.05)	−6.30 (0.08)	−6.65 (0.06)	−6.85 (0.07)
−7	83.00	−6.43 (0.06)	−6.74 (0.05)	−7.00 (0.09)	−7.22 (0.02)	−7.81 (0.13)	−7.99 (0.13)
−8	93.61	−7.24 (0.03)	−7.74 (0.20)	−8.00 (0.08)	−8.34 (0.05)	−8.82 (0.08)	−9.14 (0.07)
−9	105.47	−8.22 (0.03)	−8.44 (0.06)	−9.02 (0.11)	−9.37 (0.12)	−10.05 (0.07)	−10.33 (0.08)

*WP values under each temperature column represent mean and (SD) measured by WP4C*.

a *PDB and agar added at 24 and 20 g per 1,000 ml of water at 20°C, respectively*.

**Table 4:** Water potential (MPa) of KCl amended potato dextrose agar (KCl+PDB+agar).

**Target WP**	**KCl conc. (g/l PDA^a^)**	**7°C**	**15°C**	**20°C**	**25°C**	**30°C**	**35°C**
0	0.00	−0.31 (0.01)	−0.55 (0.07)	−0.55 (0.01)	−0.46 (0.01)	−0.46 (0.04)	−0.50 (0.05)
−1	14.55	−0.91 (0.01)	−1.23 (0.10)	−1.00 (0.04)	−1.06 (0.04)	−1.23 (0.03)	−1.44 (0.09)
−2	31.73	−1.77 (0.05)	−1.96 (0.10)	−2.00 (0.02)	−2.14 (0.03)	−2.32 (0.03)	−2.51 (0.08)
−3	48.36	−2.71 (0.01)	−2.89 (0.08)	−2.99 (0.05)	−3.11 (0.03)	−3.36 (0.03)	−3.55 (0.05)
−4	66.27	−3.76 (0.03)	−3.96 (0.04)	−3.99 (0.04)	−4.20 (0.05)	−4.52 (0.09)	−4.76 (0.02)
−5	80.45	−4.58 (0.04)	−4.78 (0.10)	−5.01 (0.04)	−5.20 (0.03)	−5.48 (0.06)	−5.68 (0.02)
−6	96.71	−5.53 (0.03)	−5.73 (0.06)	−6.01 (0.04)	−6.24 (0.04)	−6.56 (0.05)	−6.76 (0.03)
−7	114.06	−6.43 (0.05)	−6.64 (0.11)	−7.03 (0.04)	−7.29 (0.01)	−7.76 (0.07)	−8.04 (0.04)
−8	130.36	−7.47 (0.06)	−7.69 (0.13)	−8.06 (0.04)	−8.32 (0.02)	−8.91 (0.04)	−9.17 (0.07)
−9	145.79	−8.19 (0.01)	−8.44 (0.20)	−9.03 (0.04)	−9.33 (0.03)	−9.94 (0.06)	−10.24 (0.06)
−10	159.84	−9.01 (0.03)	−9.15 (0.10)	−10.01 (0.09)	−10.24 (0.06)	−10.91 (0.07)	−11.29 (0.05)

*WP values under each temperature column represent mean and (SD) measured by WP4C*.

a *PDB and agar added at 24 and 20 g per 1,000 ml of water at 20°C, respectively*.

WP4C uses the chilled-mirror dew-point technique which integrates both osmotic and matric components of WP of a sample. Since the machine has internal temperature control from 15 to 40°C, sample WP from 15 to 35°C was determined on lab benchtop. For determining WP of a sample at 7°C, the temperature control of the device was turned off, and the machine was placed in an incubator at 7°C. Though WP4C has a range of measuring WP from 0 to −300 MPa with a very high accuracy, like all vapor pressure instruments its accuracy decreases in the wet end of the WP range. Samples wetter (less negative) than −1 MPa were read in continuous mode in stainless steel cups to improve the accuracy in wet range. All other samples drier (more negative) than −1 MPa were read in the precise mode in disposable plastic sample cups.

For aqueous solutions of PDB, 24 g of PDB (Difco, MD, USA) was dissolved in 1 liter of deionized water and amended with a variable amount of PEG-8000 (EMD Chemicals, Inc. USA) (Table 1) or NaCl (Fisher Scientific, USA) (Table 2). For PDA, 24 g of PDB and 20 g of agar (Sigma-Aldrich, Co. USA) were dissolved in 1 liter of deionized water and amended with a variable amount of NaCl (Table 3), or KCl (Avantor Performance Materials, Inc. USA) (Table 4).

After dissolving, all salt-amended media combinations were autoclaved at 121°C for 30 min followed by cooling and pouring in petri plates (80 mm dia.) under running air in a laminar flow to simulate microbiological inoculation procedures. Care was taken to mix the media well with any condensed water on the walls of autoclaving flasks before pouring into petri plates. Media in petri plates was equilibrated for 24 h at the same temperatures in the incubator at which the readings were to be taken on WP4C.

WP of these autoclaved, salt-amended media combinations was then determined on WP4C. The volume of PDB and the chunk size of solid PDA removed from the middle of the petri plate was large enough to cover the surface of sample cup.

All statistical tests and model fitting were done using statistical software R 3.3.2. (R Core Team, 2016). A null model with no parameter and, a global model of third-order interactions between temperature and solute concentration was set up. Model selection was made by Akaike Information Criterion (AIC) in a stepwise algorithm in both directions given in “MASS” package (Venables and Ripley, 2002). Parsimonious model with higher predictability power was the basic criterion. Parameter importance in a model was cross-checked with Bayesian Information Criterion (BIC). Any parameter not appearing significant in BIC was further removed from the model selected by forwarding stepAIC.

## Results

### PDB amended with PEG

At any given concentration of PEG, the WP of PEG-amended PDB increases (becomes less negative) with the rising temperature (Table 1). For example, 584 g of PEG in PDB is required to attain a target WP of −5 MPa at 20°C. However, with this same concentration, the WP value is −5.61 and −3.97 MPa at 7 and 35°C respectively. For successive WP levels, the incremental PEG concentration declines in batches after the initial steep decrease between −1 and −2 MPa. The range of WP between 7 to 35°C also increases with each successive WP level.

### PDB amended with NaCl

With 0 g of NaCl, WP of basal PDB ranged from −0.26 to −0.49 MPa, with no clear trend with temperature (Table 2). With the addition of NaCl, the WP of amended PDB decreased (became more negative) with the increase in temperature. The range of WP between 7 and 35°C increased incrementally with increasing concentrations of NaCl, from 0.08 MPa where the target was −1 MPa to 1.11 MPa where the target was −9 MPa. But the incremental NaCl concentration and ratio of the deviation to the targeted WP remains relatively constant with successive WP levels.

### PDA amended with NaCl or KCl

Similar to basal PDB, the WP of basal PDA did not show a clear trend with temperature. The range of WP from 7 to 35°C also remained the same, but the WP values were more negative in basal PDA than basal PDB for the same temperature (Table 3).

With the addition of NaCl or KCl in basal PDA, WP became more negative with the increasing temperature and concentration (Tables 3, 4). The trend is the same as seen in NaCl amended PDB, but the range of the deviation from 7 to 35°C is more in PDA than PDB with successive WP levels.

### Models for predicting water potential from temperature and solute concentration in PDB and PDA

Besides being parsimonious, the model selection was based on their prediction accuracy. The accuracy criterion was set at ±0.05 to 0.10 MPa deviation of model-predicted values from WP4C observed values at given temperature and solute concentration. These values are based on the accuracy range of the dew point hygrometer (WP4C) used in this study. This approach was necessary because otherwise even the linear models with one (solute concentration) or two (solute concentration and temperature) explanatory variables had high adjusted *R*^2^-values (>0.90), but low prediction accuracy. For example, in NaCl-amended PDB, the adjusted *R*^2^ of a linear model for predicting WP from NaCl concentration alone and, NaCl concentration and temperature was as high as 0.9938 and 0.9966, respectively but their prediction accuracy was below 32 percent. The adjusted *R*^2^ of NaCl-amended PDB model presented in Table 5 is 0.9996 with a prediction accuracy of 93 and 98 percent at 0.05 and 0.10 MPa deviation levels, respectively.

**Table 5:** Model parameters relating water potential of potato dextrose broth and potato dextrose agar to temperature and salt concentration.

**Coefficient^a^**	**Potato Dextrose broth^b^**		**Potato Dextrose agar^c^**	
	**PEG**	**NaCl^d^**	**NaCl**	**KCl**
β_0_	−3.931e-01	7.204e-02	−3.474e-01	−3.908e-01
β_1_	3.376e-03	−6.942e-02	−4.726e-02	−3.996e-02
β_2_	–	−7.238e-02	−4.459e-03	–
β_3_	−2.583e-05	−8.771e-05	−5.471e-04	−2.540e-04
β_4_	–	3.412e-03	–	–
β_5_	9.412e-09	3.622e-07	2.502e-06	9.926e-07
β_6_	–	−4.975e-05	–	−2.954e-06
β_7_	−2.937e-04	–	–	4.036e-04
β_8_	3.168e-07	−4.655e-06	–	5.010e-06
β_9_	−1.611e-10	–	9.035e-08	−2.399e-08
β_10_	1.388e-05	–	−2.501e-05	−5.242e-05
β_11_	–	–	3.044e-07	–
β_12_	–	3.438e-10	−8.081e-09	−
β_13_	−2.061e-07	–	–	7.293e-07
β_14_	–	–	1.047e-10	–
adj. *R*^2^	0.9993	0.9996	0.9988	0.9991

*Formula ^a^: WP = β_0_ + β_1_x + β_2_y + β_3_x^2^ + β_4_y^2^ + β_5_x^3^ + β_6_y^3^ + β_7_xy + β_8_x^2^y+ β_9_x^3^y + β_10_xy^2^ + β_11_x^2^y^2^ + β_12_x^3^y^2^ + β_13_xy^3^ + β_14_x^3^y^3^, where WP is water potential in MPa, x is PEG or NaCl or KCl in gram per liter of PDB or PDA in water and y is temperature in °C*.

a *The general formula of model will be reduced depending on the number of coefficients listed in each solute column*.

b *For basal PDB (0 g PEG/NaCl), use coefficients listed under NaCl in PDB*.

c *For basal PDA (0 g NaCl/KCl), use this model: WP = −0.1620677 −0.0302276y + 0.0006286y^2^*.

d *p-values highly significant for all coefficients except intercept in NaCl amended PDB*.

Simpler models were also deficient in accounting for higher order relations and interactions between WP, solute concentration, and temperature. For example, in PEG-amended PDB, the five-parameter quadratic model with a single order interaction between PEG and temperature has a prediction accuracy of only 26 and 58 percent at 0.05 and 0.10 MPa deviation levels, respectively while the nine-parameter model given in Table 5 improves this accuracy to 73 and 95 percent, respectively. Alternately, the range of standard deviation is reduced from −0.27 to 0.30 MPa (five-parameter model) to −0.12 to 0.14 MPa (nine-parameter model). Similarly, in NaCl-amended PDA, the prediction accuracy of the nine-parameter model (Table 5) is 70 and 82 percent compared to 52 and 75 percent in a five-parameter quadratic model with interaction at 0.05 and 0.10 MPa deviation levels, respectively.

Despite considering these facts in the final model selection, only NaCl and PEG-amended PDB model have >95 percent prediction accuracy at the maximum set deviation level of 0.10 MPa. For NaCl and KCl-amended PDA, prediction accuracy was 82 and 85 percent, respectively at the 0.10 MPa deviation level. This lower efficiency in PDA models is due to larger deviations of predicted vs. observed WP values at lower dilution levels. For these two PDA models, further improvements in the prediction require the inclusion of more parameters, but the marginal gains are not high.

With no added solute in basal PDB and PDA, their WP is best described by temperature alone quadratic models. The absence of required temperature parameters in the NaCl and KCl-amended PDA models make them deficient in predicting WP of basal PDA. This disability of PDA models given in Table 5 with basal PDA level necessitated a separate quadratic model based on the relation of basal PDA (zero salts) WP to the temperature given in the footnote of Table 5.

### Synergy level between NaCl, PDB, and agar

**Figure 1:**
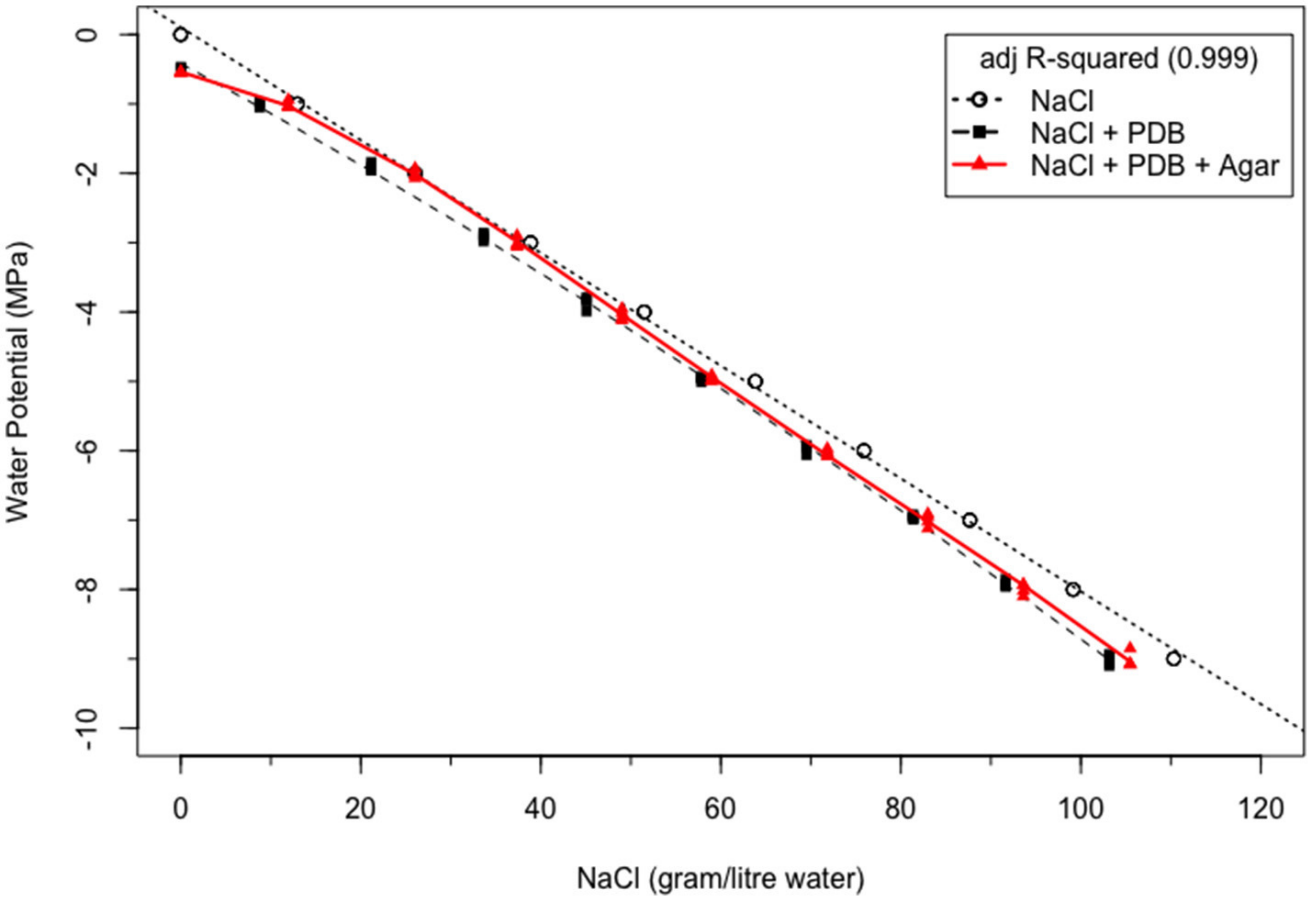
Synergism between NaCl, potato dextrose broth and agar in NaCl-amended potato dextrose broth, NaCl amended potato dextrose agar and pure NaCl solution at 20°C.

In Figure 1, the NaCl concentration required to attain WP levels from 0 to −9 MPa at 20°C for NaCl solution were calculated from Lang (1967). The relationship between WP and NaCl concentration was linear (dotted line). In PDB, less NaCl was required to attain the same WP level at 20°C, and the relation between WP and NaCl in PDB was quadratic (dashed line). But when agar was introduced as a third component in the same NaCl amended PDB at the same temperature of 20°C, more NaCl was required to attain the same WP level in agar + PDB vs PDB alone. The relation between WP and NaCl concentration in PDA was best explained by the cubic model (red solid line). In comparing PDB + agar to NaCl alone, less NaCl was needed in the former at WP's less than ≤ 3 MPa and between 0 and −1 MPa. However, to attain −2 MPa, the amount of NaCl required was similar in both systems. In general, the differences between NaCl alone and with PDB and agar become greater at drier (more negative) WPs.

## Discussion

This study has demonstrated that with PDB and agar, the final WP of a solution is not an additive function of the WP of its individual components. We have accurately documented the synergy among these elements of PDB and PDA and, how temperature further influences these interactions. More importantly, we have derived accurate models that can be used for designing experiments to measure how WP affects fungal growth.

For pure solutions of NaCl or KCl, the relation between WP and temperature, and WP and concentration are linear (Lang, 1967; Campbell and Gardner, 1971). The WP of PEG solutions, vary quadratically with concentration and linearly with temperature (Michel and Kaufmann, 1973). But in solute-amended PDB or PDA, the relationship between WP and the temperature is best described by quadratic models. Within each temperature, the relationship between WP and concentration of NaCl or KCl in PDA and PEG in PDB are best described by cubic models, and by the quadratic fit between WP and NaCl concentration in PDB. The temperature in interaction with solute concentration influences the resultant WP for any media combination.

NaCl in PDB and NaCl or KCl in PDA follow the general trend of increasing negativity of WP values with increasing temperature. But in PEG amended PDB, WP value becomes less negative with increasing temperature. The reason for this is decreasing viscosity with the increase of temperature (Michel and Kaufmann, 1973). Less negative WP means more free water is available for uptake by a microorganism.

Each added increment of any salt (NaCl, KCl, or PEG) not only lowers the WP but also broadens the range of variation between 7 and 35°C. While the incremental NaCl weight needed in PDB and PDA to adjust for temperature with successive WP levels varies only by 0–4 g, PEG incremental weight need declined with successive WP levels after the initial sharp drop between −1 and −2 MPa. Michel and Kaufmann (1973) have attributed this declining incremental PEG weight to increased viscosity with the increase in concentration.

The studies conducted by Lagerwerff et al. (1961), Michel and Kaufmann (1973), Michel (1983), and Money (1989) proved that the WP of individual components cannot be directly added to calculate the whole WP of any PEG solution having other solutes. Total WP of this multi-solute PEG solution will alter because of positive or negative synergism between its constituents. The addition of agar in basal PDB (zero salts) makes the WP of basal PDA more negative than basal PDB. This decreasing WP indicates positive synergy between PDB and agar. Agar itself contributes to basal media WP, and any increase in agar concentration decreases basal media WP (Buah et al., 1999). PDB also has positive synergy with NaCl. Lower NaCl concentration is required in PDB to achieve the same level of WP as in true NaCl solution. But the addition of agar to the existing solution of NaCl amended PDB increased the amount of NaCl to reach the same WP levels. It indicates that despite the combination of PDB and agar, and PDB and NaCl having positive synergy among themselves, the combination of all three in NaCl amended PDA rather decreases synergism.

Moreover, synergism is dynamic and changes with the change of temperature (Michel, 1983). The accuracy criterion of ±0.05–0.10 MPa deviation between the predicted and the observed WP values employed in the model selection for solute-amended PDB and PDA takes care of this temperature influenced synergy dynamics between its components. These models will hold if PDB and agar concentrations remain same.

Autoclaving is another variable that we took into consideration, given that all growth media need to be sterilized before use. Van der Weele et al. (2000) have stated 4 and 20% decrease in WP of an autoclaved −1.6 MPa NaCl and PEG solution, respectively. In the current study, all WP standardizations were done after autoclaving salt-amended media. Also, the methodology followed for media plating and reading in WP4C simulates the plating and inoculation steps in microbiological studies. So, these WP values can be used in studying the biology of all microorganisms culturable on PDB and PDA in the laboratory.

Besides providing actual WP values, the findings of this research show the impact of temperature and discrepancies arising due to the level of synergism between various constituents of a growth medium. A simple addition of the separate values of WP for the unamended nutrient solution and the solute in distilled water may not reflect the whole WP of the medium.

## Author contributions

TP conceived and obtained funding for the research and edited paper. IA carried out research, did statistical analysis and wrote paper. This work was done as part of the Ph.D. thesis of IA. He designed and conducted all the experiments, collected and analyzed the data, and wrote the drafts of the manuscript. The research plans and analysis were done in collaboration with TP. TP was the PhD thesis supervisor.

### Conflict of interest statement

The authors declare that the research was conducted in the absence of any commercial or financial relationships that could be construed as a potential conflict of interest. The reviewer JMA and handling Editor declared their shared affiliation, and the handling Editor states that the process nevertheless met the standards of a fair and objective review.
